# Applying the resist–accept–direct (RAD) framework to wildlife health management

**DOI:** 10.1093/biosci/biaf061

**Published:** 2025-06-12

**Authors:** Wynne E Moss, Gregor W Schuurman, Emily S Almberg, Danielle E Buttke, Nathan L Galloway, Samantha E J Gibbs, Anne Hubbs, Katherine L D Richgels, C LeAnn White, Paul C Cross

**Affiliations:** U.S. Geological Survey, Northern Rocky Mountain Science Center, Bozeman, Montana, United States; National Park Service's Climate Change Response Program, Fort Collins, Colorado, United States; Montana Fish, Wildlife & Parks, Bozeman, Montana, United States; National Park Service's One Health Program, Fort Collins, Colorado, United States; National Park Service's Biological Resources Division, Fort Collins, Colorado, United States; U.S. Fish & Wildlife Service's National Wildlife Refuge System, Chiefland, Florida, United States; Alberta Environment and Protected Areas, Rocky Mountain House, Alberta, Canada; U.S. Geological Survey National Wildlife Health Center, Madison, Wisconsin, United States; U.S. Geological Survey National Wildlife Health Center, Madison, Wisconsin, United States; U.S. Geological Survey, Northern Rocky Mountain Science Center, Bozeman, Montana, United States

**Keywords:** wildlife health, wildlife conservation, natural resource management, resist–accept–direct (RAD) framework, infectious diseases, emerging diseases

## Abstract

Wildlife diseases can have substantial impacts on wildlife populations as well as on human and domestic animal health and well-being. Although many agencies and stakeholders share a goal of supporting wildlife health, reducing wildlife disease burden is complicated by a scarcity of effective interventions for wildlife, competition for funds, and conflicting priorities. As a result, agencies are unlikely to avoid the impacts of wildlife diseases in all contexts and need to evaluate where resisting disease is most feasible and beneficial. The resist–accept–direct (RAD) framework is a tool that assists natural resource managers in exploring and communicating about management interventions, including in situations where resisting ecological changes may not be possible. In the present article, we discuss how the RAD framework could be adapted to wildlife disease contexts to address several outstanding challenges in wildlife health management.

Infectious diseases can have substantial consequences for wildlife populations and biodiversity, with impacts extending beyond natural ecosystems to agricultural and human health, as well as the sociocultural values and services offered by wildlife (Delahay et al. 2009, Smith et al. 2009). As a result, interest in wildlife disease management is shared by natural resource, public health, and agricultural sectors, which expend considerable effort to mitigate disease spread and impacts (Gortazar et al. 2015, Portier et al. 2019, Sokolow et al. 2019).

Despite wide interest, effective wildlife disease management remains elusive. Many wildlife disease management techniques were derived from the veterinary, agricultural, and public health fields and are ineffective or impractical for wildlife populations (Woodroffe 1999, Sokolow et al. 2019). Where effective strategies exist for controlling pathogens or disease in wildlife (e.g., depopulation, biosecurity), social acceptability or a lack of compliance often limits their implementation (Leong and Decker 2020). Logistical challenges, governance issues, and resource constraints may further limit application of interventions at relevant scales or on desired timeframes (Grant et al. 2017). Important knowledge gaps for many wildlife diseases compound these difficulties (Joseph et al. 2013, Grant et al. 2017). Even for the small minority of diseases that have received widespread attention and significant funding (e.g., chronic wasting disease, white-nose syndrome, chytridiomycosis), the diseases continue to spread and drive population declines or other negative impacts (Scheele et al. 2019, Thompson et al. 2023, Hadlock and Kaup 2024). At the same time, species range shifts, climate change, global trade, agricultural practices, and biodiversity loss have promoted the emergence of novel and high-impact wildlife diseases, placing pressure on agencies to manage an ever-growing list of pathogens with limited resources (Mahon et al. 2024). In a world with rapidly changing disease risk, current management efficacy is also shifting, and agencies may need to reconsider where and how to intervene or whether to cease control efforts altogether.

The resist–accept–direct (RAD) framework arose in response to a similarly challenging problem in natural resource management: Ecosystem transformation under directional climate change and the realization that maintaining historical ecological conditions (i.e., *resisting* change) was not always feasible (Schuurman et al. 2020, Lynch et al. 2021, Thompson et al. 2021, 2022). The RAD framework describes three potential responses to ecosystems undergoing substantial change (Schuurman et al. 2020). Managers can choose to *resist* the change and attempt to maintain historical or currently acceptable conditions, to *accept* the change and allow ecosystems to drift into new conditions, or to actively *direct* the system toward novel states that support preferred services and conditions (table 1). RAD approaches are defined by the intended outcome of a management action (preservation of historical conditions or not) and the degree of management intervention required (figure 1; Schuurman et al. 2022). The framework can support a flexible approach to natural resource decision-making that is dynamic across both space and time. For example, agencies may use a portfolio of RAD strategies such as *resisting* changes in certain populations, biological scales, or ecosystem properties while *accepting* changes in others (figure 2). The RAD framework is also generally applied within an adaptive management context that encourages revisiting decisions in response to new information or changes in values or policy (Lynch et al. 2022, Magness et al. 2022).

**Figure 1.**
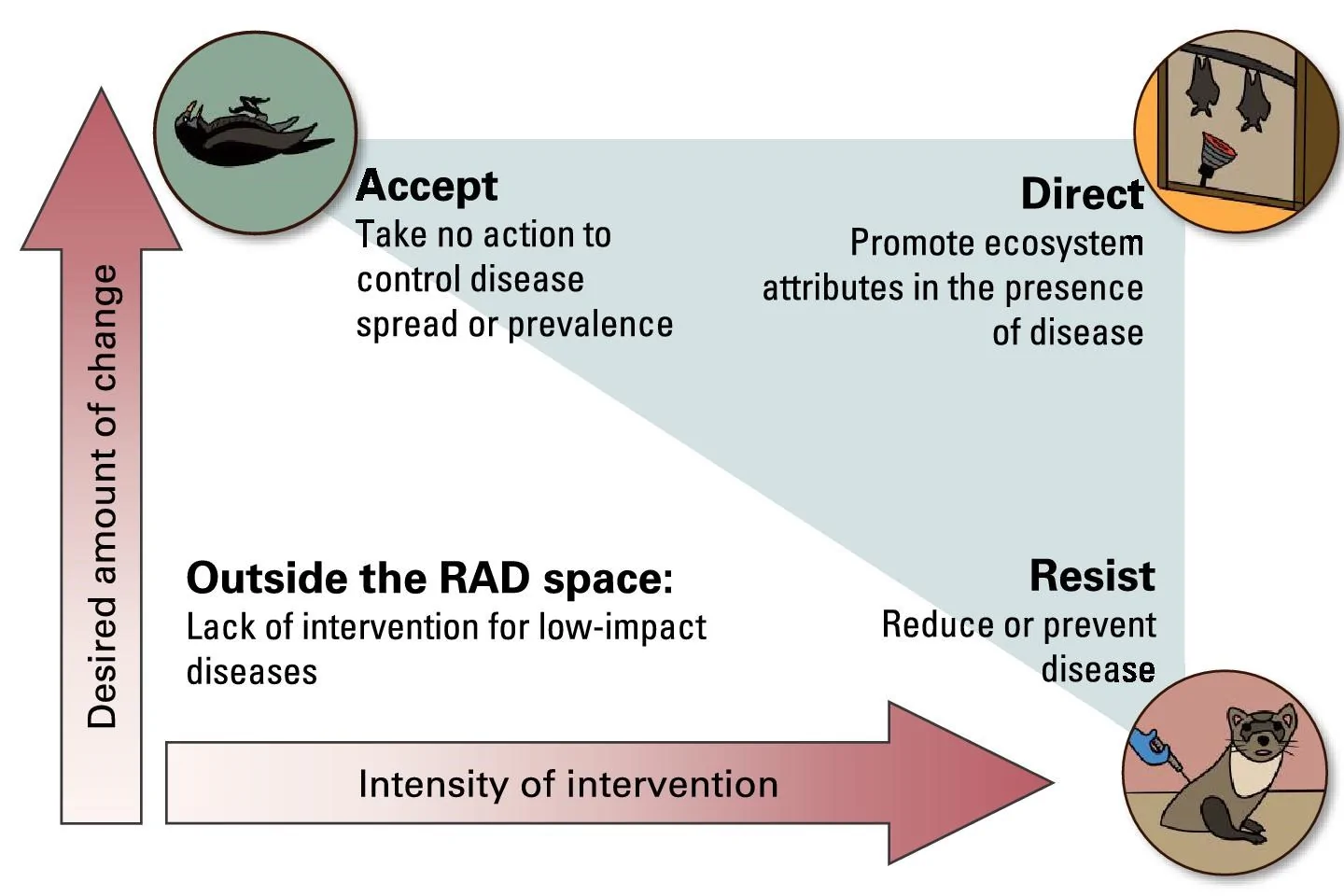
RAD (resist–accept–direct) decisions are characterized by the amount of management intervention required and the degree of ecological change sought by a management intervention. Source: The figure was adapted from Schuurman and colleagues (2022). Examples of resist, accept, and direct actions on wildlife disease include vaccination and reintroduction of black-footed ferrets to establish disease-free populations, acceptance of West Nile virus spread in some avian populations, and supporting white-nose syndrome-affected bat populations using heated bat boxes, respectively.

**Figure 2.**
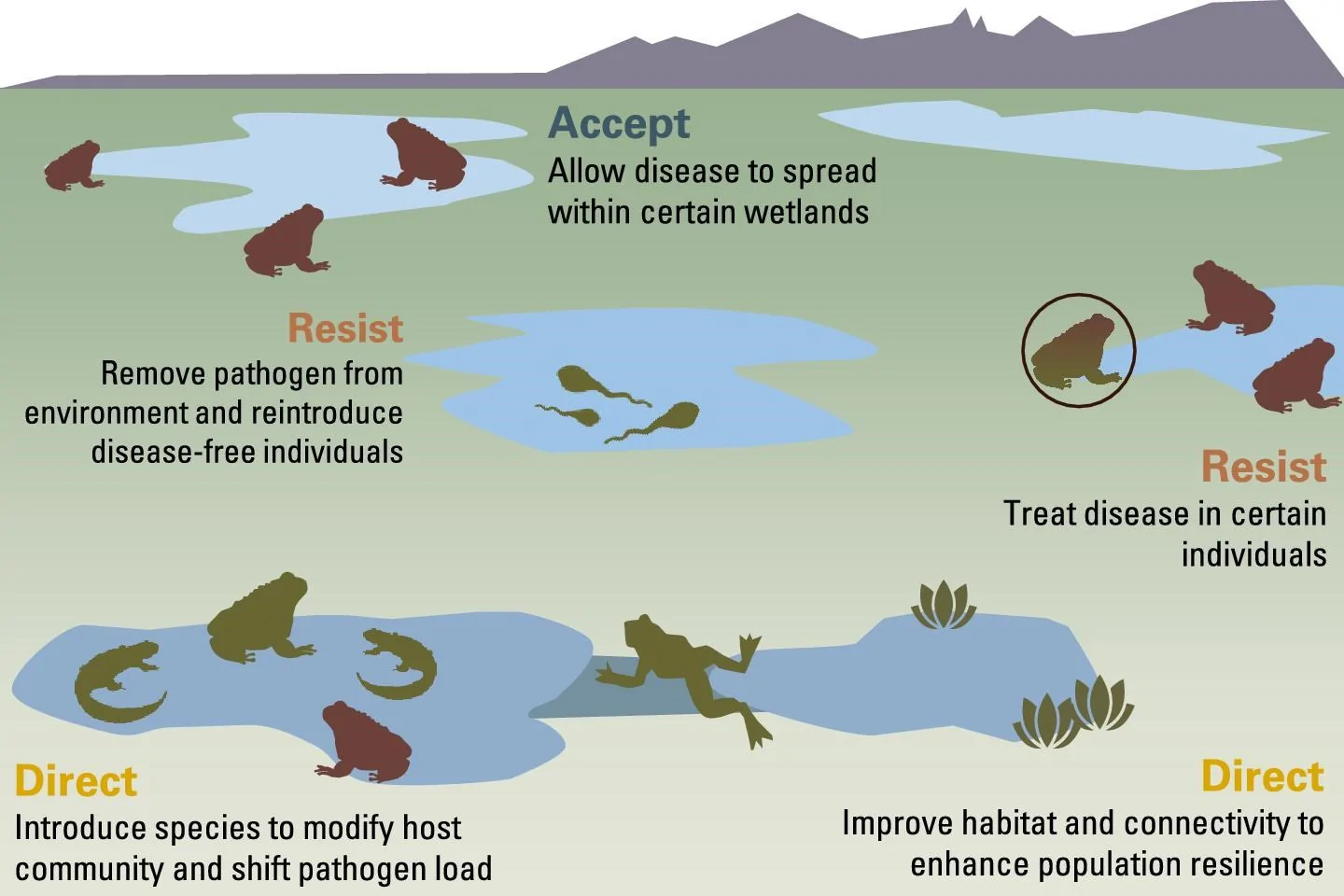
RAD (resist–accept–direct) approaches can be used dynamically across space and scale. Managers may resist, direct, or accept disease in certain individuals, populations, or geographic locations. A portfolio of approaches may be deployed, as is depicted in the figure for a hypothetical amphibian disease within a complex of wetlands. Source: The images are original or from www.phylopic.org (salamander: Matt Reinbold, modified by T. Michael Keesey).

**Table 1.** The resist–accept–direct framework for natural resource management.

**Framework element**	**Resist**	**Accept**	**Direct**
Original definition	Maintain or restore ecosystem composition, structure, or function on the basis of historical or acceptable current conditions	Allow ecosystem composition, structure, and function to drift autonomously (away from historical conditions)	Actively shape ecosystem composition, structure, and function to create a new ecosystem configuration on the basis of preferred conditions and ecosystem services
Application to disease	Reduce or prevent disease	Take no action to control disease spread or reduce prevalence	Promote preferred ecosystem attributes in the presence of disease by actively altering disease risk or impact
Desired outcomes	Eradication of disease at targeted scalePrevention of disease spreadElimination of disease impacts and return to pre-emergence conditions	Use of limited resources on other management needsNatural evolution of pathogens or hosts	Coexistence between wildlife and pathogensWildlife population persistence
Example interventions	Biosecurity measuresTreatments or vaccinationsRemoval of infected individuals	No specific interventions neededMay involve actions such as monitoring or communication	Enhancing adaptive capacity to disease through facilitated evolution Reductions of additional stressors on populations

*Note:* This table describes original definitions for each category, adapted from Schuurman and colleagues (2020) and Lynch and colleagues (2021), and how the framework could be adapted for wildlife disease management.

The RAD framework has helped agencies across a variety of settings consider a wider range of management responses to ecosystems affected by climate change and other stressors (e.g., US National Park Service 2021, Wilkening et al. 2022, Ward et al. 2023, Olsson et al. 2024). However, application of the RAD framework to wildlife disease is not yet common practice. In the present article, we illustrate the relevance of the RAD framework for decision making surrounding wildlife health, discuss possible interpretations of the framework in wildlife disease contexts, and highlight challenges and considerations.

## The RAD framework for wildlife health

Wildlife management agencies already regularly make decisions about where and how to intervene in disease outbreaks (Wobeser 2002, Delahay et al. 2009, Portier et al. 2019). These decisions are often guided by existing frameworks and decision support tools (e.g., adaptive management, scenario planning, disease risk assessments, structured decision-making) and draw on a diverse and growing toolbox of interventions (Wobeser 2002, Langwig et al. 2015, Grant et al. 2017). The RAD framework is not intended to replace these processes or introduce new methods for disease control or treatment. Instead, the framework is a tool for broadening the suite of options considered in natural resource management and adding clarity, pragmatism, creativity, and intentionality to management decisions (table 2). The selection and implementation of specific interventions may draw on additional decision support tools and be supported by activities such as monitoring and evaluation within an adaptive management framework (Lynch et al. 2022, Magness et al. 2022).

**Table 2.** The benefits of resist–accept–direct (RAD) thinking.

**Benefit**	**Description**
Avoiding default decision making	RAD encourages transparent and intentional decision-making, rather than implementing strategies as a default or reflexive action.
Using a shared language	As RAD becomes increasingly common, use of RAD in wildlife health promotes a shared language and philosophy across natural resource contexts.
Clarifying goals	Selecting a RAD strategy encourages agencies to formalize and communicate the goal of a given management action.
Allocating resources efficiently	RAD can prompt decision-makers to question strategies that are inefficient or overly resource-intensive.
Thinking outside the box	Use of RAD can shift existing paradigms and entrenched ideas about wildlife health management, leading to the generation of new and creative solutions.
Acknowledging reality	Exploring the RAD space helps managers confront the possibility that resistance is not always possible (or desirable).
Emphasizing wildlife health	The RAD framework prompts agencies to consider other goals besides reducing disease, leading to a more holistic vision of wildlife health.

In the following sections, we suggest interpretations for each RAD category, based on our experiences working at the intersection of disease ecology and wildlife management and focusing primarily on wildlife health management by natural resource management agencies (rather than public health or agricultural agencies). As RAD is applied to a growing number of natural resource challenges (e.g., invasive species management, Dunham et al. 2022; endangered species recovery, Schuurman et al. 2023; coral bleaching, Rogers‐Bennett et al. 2022), users have adapted its definitions for each unique context. Therefore, we emphasize that our interpretations should be viewed as an illustration of RAD's applications to wildlife health and not a universal set of definitions.

## Resist

In the context of wildlife health, we suggest that *resisting* is the aim of maintaining or restoring wildlife to a disease-free condition. This could entail keeping wildlife free from the focal pathogen (i.e., achieving a prevalence of zero) or eliminating disease symptoms through complete treatment. Alternatively, *resisting* could entail reducing disease prevalence below a level where any population impacts are seen, thus aiming to return focal wildlife populations to a desired pre-disease-emergence state. Agencies may *resist* disease at a variety of scales, ranging from specific individuals, locations, or population units all the way up to entire species, jurisdictional units, or countries (figure 2). Although the implementation scale may differ, the common goal with *resist* strategies is the prevention of disease or disease impacts at the chosen scale of management.

*Resisting* disease often involves preventative strategies (e.g., biosecurity measures) intended to avoid infectious agent introduction to naive populations. Preventative measures are considered the most effective strategies for maintaining disease-free populations and are actively encouraged across a range of contexts (Langwig et al. 2015). For example, bans on wildlife importation and transport have thus far been effective at avoiding salamander chytrid fungus (*Batrachochytrium salamandrivorans*) introduction to North America (Waddle et al. 2020). Decontamination protocols and restricted access are used to prevent human-assisted introduction of the fungal pathogen *Pseudogymnoascus destructans* (the causative agent of white-nose syndrome) to naive bat populations (Shapiro et al. 2021). *Resist* strategies may also involve vaccination or treatment programs that seek to eradicate or prevent disease. Mass vaccination campaigns are relatively rare in wildlife but have been employed in some populations, such as reintroduced black-footed ferrets (*Mustela nigripes*), which are commonly vaccinated against plague (Salkeld 2017). Disease eradication or prevention can also include environmental treatment or modification to remove vectors or infectious pathogens. For instance, wetland management (e.g., hydroperiod modification, predator introduction) is used to suppress mosquito breeding and prevent mosquito-borne disease (Rey et al. 2012). Chemical treatments have also been used to eliminate vectors or pathogens at certain locations (e.g., chytridiomycosis; Bosch et al. 2015). Finally, agencies may *resist* disease using selective removals or population culls. Applications include test and cull for bighorn sheep (*Ovis canadensis*) pneumonia (Garwood et al. 2020) and white-tailed deer (*Odocoileus virginianus*) and reindeer (*Rangifer tarandus*) culls for chronic wasting disease (Thompson et al. 2023).

A decision to *resist* may be particularly desirable for diseases that affect highly imperiled species or that have spillover potential or for which effective strategies already exist and are easily implemented. A focus on *resisting* disease is often promoted during early disease stages, when efforts have a higher chance of success (Langwig et al. 2015). Indeed, the idea that agencies should attempt to prevent novel disease establishment is pervasive and has parallels with invasive species management (i.e., early detection, rapid response; Reaser et al. 2020). In practice, however, attempts to *resist* disease can be ineffective, particularly if the disease is already well established (Bozzuto et al. 2020), and may involve important trade-offs. In some contexts, interventions such as vaccines, culls, or pharmaceutical treatments can increase disease transmission or worsen adverse impacts on populations (Gortazar et al. 2015, Barnett and Civitello 2020). For instance, population culls aimed at reducing the density of reservoir hosts can result in shifts in wildlife movements, facilitating disease spread to new areas (e.g., rabies, Viana et al. 2023; bovine tuberculosis, Donnelly et al. 2006). Moreover, a lack of knowledge, resources, stakeholder support, or agency prioritization often limits action during critical early windows, and agencies often attempt to manage diseases long after containment efforts have failed (Langwig et al. 2015, Bernard and Grant 2019). Importantly, ineffective disease eradication or prevention efforts can erode stakeholder confidence in an agency's ability to manage disease and reduce support for the management activity itself (Holsman et al. 2010). This is particularly true when interventions to resist disease entail significant financial or social costs. Another important consideration is that *resisting* disease often requires sustained intervention to counteract the constant threat of disease invasion and evolution (Wobeser 2002).

In summary, although *resist* strategies may work during certain disease stages or for particular pathogens, judicious use is warranted. The common strategy of *resisting* first and then switching to other strategies may not be appropriate in all contexts. The RAD framework may help avoid investments in resistance strategies that are ineffective, resource intensive, involve unwanted trade-offs, or threaten stakeholder support. Using the RAD framework to explore alternative options early on can enable agencies in some cases to proactively allocate limited resources to more effective conservation activities.

## Accept

Natural resource managers may also consider *accepting* disease presence and impacts without intervention. To *accept* means to allow a disease to run its course autonomously; however, acceptance does not imply a total absence of action (Schuurman et al. 2022). For example, an agency that choose an *accept* strategy of ceasing attempts to control disease transmission may still decide to take actions such as communicating with the public about the disease and (change in) management approach and monitoring populations to evaluate outcomes and revisit management objectives as needed (Portier et al. 2019, Lynch et al. 2022). Decisions to *accept* disease spread can be applied selectively to some populations or species while still resisting or directing diseases in others. For instance, agencies may *accept* disease spread within certain wildlife species but still aim to resist spillover to livestock or humans by reducing contact rates or animal movement at important interfaces (Sokolow et al. 2019).

Several factors may contribute to a decision to *accept*. First, certain hosts or pathogens may be more amenable to the *accept* strategy. For instance, managers may decline to intervene when diseases affect wildlife species with wide distributions or with high adaptive capacities or where the pathogen poses little risk to humans or livestock. Agencies may also opt to cease costly or time-intensive interventions, such as those involved with resisting or directing disease, to divert resources to other high-priority management activities. Another potential motivation for *accepting* disease spread is the desire to preserve the natural dynamics between hosts and pathogens. For example, diseases play important roles in evolutionary processes such as adaptive evolution and selection for genetic diversity and are involved in ecological processes like energy transfer and intraguild competition (Tompkins et al. 2011). The outcomes of unmanaged disease spread may range from extinction (e.g., in Australian frog species affected by chytridiomycosis; Scheele et al. 2017) to more tolerable outcomes, such as population bottlenecks followed by herd immunity (e.g., in common eiders, *Somateria mollissima*, after an avian cholera outbreak on Mitivik Island, Nunavut, in Canada; van Dijk et al. 2021). The degree to which anthropogenic factors have exacerbated disease spread and impacts may influence whether agencies decide to intervene or whether they regard a disease outbreak as “natural” (Buttke et al. 2021)—although the influence of anthropogenic factors is often challenging to assess.

A tempting strategy might be to *resist* diseases that have high impacts and *accept* those that do not. However, diseases with very little potential for impact (e.g., those with low virulence or a low likelihood of long-term persistence) likely do not require managers to use the RAD framework at all (see figure 1). Instead, the RAD framework is most useful for particularly challenging natural resource problems involving risk of substantial ecological change (Schuurman et al. 2022). The RAD framework acknowledges that agencies may *accept* ecological changes even when such changes are strongly undesirable. For example, limited resources, crisis fatigue, an absence of effective disease control strategies, or a lack of stakeholder support have led some agencies to take no actions to control even high-impact diseases such as West Nile Virus and chronic wasting disease. However, a decision not to intervene may not be outwardly acknowledged or may represent a last resort only after other strategies have failed and significant resources have been expended (Cravens et al. 2024). It is not always clear whether a lack of intervention is a purposeful action or an indication that a decision has not yet been made. Under a RAD-informed decision-making process, agencies are encouraged to be intentional and transparent about decision to take no action, which may facilitate trust and openness and avoid a perception of negligence (Schuurman et al. 2020, Dunham et al. 2022).

## Direct

Rather than choosing to prevent disease entirely or accept disease spread without intervention, agencies can also take actions to actively shift disease risk or impacts, while still allowing the disease's presence in a population. By *directing* disease spread and impacts, managers can seek to enable populations to live with and adapt to disease. Strategies used to *direct* are diverse but are distinguished from *resist* and *accept* strategies because they facilitate a larger degree of change (away from preemergence conditions) than *resist* strategies would allow (figure 1), and they are intended to produce results that are preferable or more predictable to what would happen with an *accept* strategy (Schuurman et al. 2022). Although *direct* strategies require significant management intervention (figure 1), they are typically designed to be self-sustaining, such that the amount of intervention wanes with time (Cravens et al. 2024). *Direct* strategies may result in lower disease prevalence, but this is not a requirement; rather, a *direct* approach seeks to maintain desired ecological functions in populations that have disease.

*Directing* disease may entail harm-reduction or health-promotion approaches that mitigate negative disease impacts or otherwise support and promote population resilience to better allow wildlife to persist with disease (Stephen et al. 2018). For example, creating or modifying roosting habitat (such as bat-friendly bridges or heated bat boxes) does not eradicate, prevent spread, or eliminate all disease impacts but can alleviate additional stressors on white-nose-syndrome-affected bat populations to help them better coexist with the disease (Holroyd et al. 2023). Other forms of *direct* could include translocations or facilitated evolution to introduce disease-adapted genotypes, individuals, or populations. Genetic engineering has been explored as a way to increase coral species’ disease resistance or tolerance (Van Oppen et al. 2015). The translocation of white-nose-resistant little brown bats (*Myotis lucifugus*) to populations in early epidemic stages is a potential tool for speeding adaptation (Langwig et al. 2017). At larger biological scales, *direct* actions could also include facilitating or creating novel host communities that are more equipped to persist with disease; for example, introducing dilution hosts or predators (figure 2) or replacing disease-sensitive species with resistant or tolerant hosts (analogous to actions taken in some forestry programs; Iverson et al. 2016). Such actions are increasingly discussed, particularly when disease intersects with climate change but, to the best of our knowledge, are still infrequently implemented. One recent example of *directing* communities toward higher disease resistance or tolerance involves reseeding oyster beds with disease-resistant species following disease-induced die-offs (Howie and Bishop 2021). Although some *direct* actions may also result in disease eradication, they do so by facilitating a significant change in community composition or population attributes rather than by specifically targeting pathogens. Given the large degree of ecological change (away from preemergence conditions) associated with such actions, we classify them as *direct* rather than *resist* strategies.

Using a *direct* strategy to promote wildlife persistence in the face of disease can shift the focus from managing disease agents to managing resilient wildlife populations. *Direct* approaches may therefore be better aligned with an increasingly widespread push to manage for wildlife health rather than against wildlife disease (Hanisch et al. 2012). Because the goals of *direct* actions can be less well defined than disease eradication, clear communication is particularly important when these strategies are selected, including setting expectations for how a given strategy is expected to influence disease prevalence and risk. The degree of ecological change that *direct* actions facilitate and the use of tools such as translocations or genetic engineering may create discomfort about unknown or novel outcomes (Kohl et al. 2019, Cravens et al. 2024); the deployment of these strategies and the development of stakeholder support will require robust science on their effectiveness and potential risks, including unexpected trade-offs.

## Challenges and opportunities

Use of the RAD framework for managing wildlife health differs in some key ways from its application to climate change adaptation. First, in climate change adaptation contexts, *resist* is commonly interpreted as an effort to halt the ecological transformations brought on by climate change, rather than preventing climate change itself (Schuurman et al. 2022). However, *resist* for wildlife health often involves managing the threat (disease) directly. Another key difference is that although climate change is largely directional, leading to greater impacts over time in the absence of adaptation, infectious diseases are often temporally dynamic, and may fade out even without intervention (May and Anderson 1979). Compared with climate change, managers may anticipate applying wildlife health interventions for only a limited amount of time—until populations evolve resistance, new treatments or vaccines are developed, or the disease becomes self-limiting. Whether this perception is true is not always clear, and agencies may wish to define milestones or time periods where alternative options are revisited. Finally, although RAD options are often deployed as a portfolio (e.g., *resisting* in some locations and *accepting* or *directing* in others; figure 2; Magness et al. 2022, Schuurman et al. 2022), wildlife movement and disease transmission may prevent a spatially structured approach. Instead, the same strategy may need to be applied across multiple jurisdictions for success, requiring additional cross-jurisdictional coordination on wildlife health RAD decision-making. These differences do not prevent the application of RAD for wildlife disease but require thoughtful interpretation of RAD definitions.

A potential point of confusion is that similar interventions may be employed to either *direct* or *resist* disease, and, therefore, the boundary between the two can appear fuzzy. For example, environmental treatments might be used to halt transmission (*resist*), or to boost survival and promote the spread of disease-adapted genotypes (*direct*; e.g., Maslo et al. 2017). Ultimately, as in climate change adaptation, the difference between *resist, accept*, and *direct* is defined by intent. Instead of the primary goal being disease prevention (i.e., *resist*), direct actions are intended to enable populations to live with disease and attempt to achieve better outcomes than would be expected if the disease were allowed to operate autonomously (i.e., *accept*). A further source of ambiguity is that many management programs involve actions to slow transmission or reduce prevalence but do not explicitly seek to eradicate disease (Langwig et al. 2015). These incomplete resistance actions are a gray area in which opportunities exist to more clearly define management goals. For example, if the goal is to reduce prevalence below a point where population impacts are observed, this action may be classified as *resist*, whereas seeking to temporarily slow disease spread to help populations evolve resistance might be classified as *direct* and would be more likely to succeed alongside efforts to support adaptive evolution. Users of the RAD framework can use these ambiguities as prompts to discuss and clarify overall goals and their long-term sustainability (table 2), which will be important for evaluating the success of interventions.

The benefits of applying the RAD framework to wildlife health are multifold (table 2). Agencies increasingly recognize the difficulty of disease prevention and eradication even while simultaneously implementing resource-intensive strategies for that purpose. The RAD framework prompts the intentional exploration of a wider range of overall management goals and approaches than often occurs, which can lead to the selection of forward-looking strategies that are better aligned with current constraints, may be more innovative and outside the box, and could in some cases free up limited resources for other activities (Schuurman et al. 2022). Exploring the full RAD decision space can provide a scaffold for having difficult but necessary conversations that challenge existing paradigms—a key piece of addressing wicked problems such as wildlife disease (Schuurman et al. 2022). Importantly, the RAD framework is intended to strengthen rather than supplant existing decision-making processes and management frameworks. For example, RAD could feed into several steps of a structured-decision-making process, such as helping to clarify fundamental objectives or diversify the management alternatives considered. Application of the RAD framework will also require addressing remaining challenges such as information gaps, misinformation, and social constraints such as institutional or cultural norms regarding wildlife health, which we discuss in the following sections.

## Information needs for RAD decision-making

Monitoring, including predecisional data collection, will be critical to applying the RAD framework for wildlife health management (Lynch et al. 2022). Disease surveillance data are likely essential inputs into RAD-based decision-making and, more broadly, any wildlife health program. Surveillance data not only help to identify management targets for *resisting* or *directing* disease (such as targeting certain species, demographic classes, or locations) but also help set expectations about the feasibility of different options (e.g., the likelihood of successful eradication) and guide when agencies may switch from one strategy to another. However, disease surveillance programs are rarely designed to quantify the impacts of disease on host populations. Combining disease surveillance data with monitoring of host populations (e.g., abundance, distribution, vital rates) and additional stressors (e.g., habitat loss, harvest) will be essential for informing RAD decision-making and assessing the impact of interventions on wildlife populations.

Implementing RAD decisions will rely on identifying effective, specific, interventions guided by knowledge about wildlife host and pathogen biology. For example, the effectiveness of *resist* strategies such as culling and vaccination depends on traits such as transmission, host behavior, and pathogen specificity. Collaboration among wildlife managers and researchers can help identify the information needs most relevant to making RAD decisions (Joseph et al. 2013). Previous work has outlined a science agenda for RAD decisions (Crausbay et al. 2022); refining this agenda for wildlife health is an important next step. However, some decisions may need to occur before additional information is available and may have to rely on extrapolation from similar diseases. For example, understanding which *resist* strategies are most or least effective for vector-borne diseases could shape early action for a novel vector-borne disease with little additional information. Developing a trait-based approach to RAD decision-making by linking RAD decisions and intervention strategies to disease traits (e.g., host breadth, transmission mode, life form) could be a fruitful avenue for future research or synthesis.

Importantly, decisions about whether, where, and how to intervene in wildlife disease outbreaks constitute interdisciplinary problems that cannot be fully informed by natural science alone (Lapinski 2015, McEachran et al. 2024). Navigating RAD decisions will require assessing impacts not only on wildlife populations but also on human health, agriculture, and cultural values. Therefore, RAD for wildlife health will also require contributions from experts in fields such as social justice, traditional ecological knowledge, public health, and economics.

## Agency and stakeholder support for RAD

Agency mandates, policies, and cultures play a strong role in RAD decision making (Clifford et al. 2022), and this is no different in the case of wildlife health. For instance, the idea of wildlife as a public trust (e.g., the North American Model of Wildlife Conservation) can constrain agencies’ ability to accept disease spread when it may have negative impacts on wildlife populations or on the public's use or enjoyment of wildlife resources (Decker et al. 2016). Policies aimed at preserving individual wildlife species (e.g., the US Endangered Species Act of 1973) may also inhibit agencies from considering the full suite of RAD options (Dunham et al. 2022), such as *accepting* disease spread and the associated impacts on federally protected species. Certain *direct* strategies, such as altering genotypes or species composition, may also violate institutional policies aimed at the preservation of species or environmental conditions. Moving forward, a more flexible interpretation and application of existing policies will be needed to accommodate RAD decision-making (Wilkening et al. 2022).

In addition to legal policies, agencies have directives, structures, and cultures that may either restrict or enable RAD decisions. Before adopting the RAD framework, agencies must consider how institutional factors, such as funding, personnel, expertise, and authority, will limit exploration of the full RAD space—and, equally important, how institutional structures and capacity could evolve to better enable RAD decision-making (Cravens et al. 2024). Concurrently, selecting among the RAD options may require questioning management norms such as a focus on early detection and rapid response. Already, many agencies are revisiting existing policies and mental models to assess when interventions are necessary and broaden the interpretation of what falls under wildlife health management (Buttke et al. 2021).

Finally, perceptions of disease and the tools used to manage it will intersect with RAD decision-making in multiple ways. Wildlife disease issues affect a wide diversity of society, including hunters, anglers and other outdoor recreationists, communities at risk from disease transmission, agricultural producers, wildlife-based industries, and conservationists (to name a few). These groups will influence and be influenced by wildlife disease management decisions. Social context is recognized as a key component of RAD application (Clifford et al. 2022) and will strongly shape wildlife health decisions, especially when diseases interact with human health, agriculture, economics, or cultural values. For example, diseases with zoonotic potential require special consideration because values and attitudes toward wildlife strongly shift when human health is threatened (Decker et al. 2010), and the public's desire to *resist* disease is likely to intensify (Buttke et al. 2015). The disease risk perceived by the public can be much higher than the actual risk, which can lead to public interest in wildlife disease management even where risk is low; this pattern has been observed for zoonotic diseases such as rabies and Lyme disease (Decker et al. 2010). In addition, many interventions that seek to *resist* or *direct* disease impacts (e.g., restrictions on animal transport, habitat closures, changes to harvest regulations) require public support or compliance, which is often a challenge (Holsman et al. 2010, Shapiro et al. 2023). On the other hand, the use of the RAD framework can also boost trust and understanding of wildlife management decisions by making the process more transparent, providing a common vocabulary about decisions, and helping set realistic expectations for the outcomes of management. Therefore, although RAD decisions may be constrained by public perceptions, the use of the RAD framework can also help enhance public trust in natural resource stewardship in a changing world.

## Conclusions

In a world with rapidly changing disease risks, agencies are redefining what wildlife health looks like, with the acknowledgement that disease reduction or eradication is not always a realistic goal. The RAD framework offers a potential path forward toward a more holistic, honest, and strategic approach to wildlife health management.

## Author Biography

Wynne E. Moss (wmoss@usgs.gov) is a biologist with the U.S. Geological Survey, Northern Rocky Mountain Science Center, in Bozeman, Montana, in the United States. Gregor W. Schuurman is an ecologist with the National Park Service's Climate Change Response Program, in Fort Collins, Colorado, in the United States. Emily S. Almberg is a disease ecologist with Montana Fish, Wildlife & Parks, in Bozeman, Montana, in the United States. Danielle E. Buttke is a veterinary epidemiologist with the National Park Service's One Health Program, in Fort Collins, Colorado, in the United States. Nathan L. Galloway is a wildlife disease ecologist with the National Park Service's Biological Resources Division, in Fort Collins, Colorado, in the United States. Samantha E. J. Gibbs is a wildlife veterinarian with the U.S. Fish & Wildlife Service's National Wildlife Refuge System, in Chiefland, Florida, in the United States. Anne Hubbs is a provincial wildlife science specialist with Alberta Environment and Protected Areas, in Rocky Mountain House, Alberta, Canada. Katherine L. D. Richgels is the ecology and epidemiology branch chief for the U.S. Geological Survey National Wildlife Health Center, in Madison, Wisconsin, in the United States. C. LeAnn White is the center director of the U.S. Geological Survey National Wildlife Health Center, in Madison, Wisconsin, in the United States. Paul C. Cross is a research wildlife biologist with the U.S. Geological Survey, Northern Rocky Mountain Science Center, in Bozeman, Montana, in the United States.
